# Immunotherapies targeting tumor vasculature: challenges and opportunities

**DOI:** 10.3389/fimmu.2023.1226360

**Published:** 2023-09-01

**Authors:** Hassan Dianat-Moghadam, Reza Nedaeinia, Mohsen Keshavarz, Mehdi Azizi, Mohammad Kazemi, Rasoul Salehi

**Affiliations:** ^1^ Pediatric Inherited Diseases Research Center, Isfahan University of Medical Sciences, Isfahan, Iran; ^2^ Department of Genetics and Molecular Biology, School of Medicine, Isfahan University of Medical Sciences, Isfahan, Iran; ^3^ The Persian Gulf Tropical Medicine Research Center, The Persian Gulf Biomedical Sciences Research Institute, Bushehr University of Medical Sciences, Bushehr, Iran; ^4^ Department of Tissue Engineering and Biomaterials, School of Advanced Medical Sciences and Technologies, Hamadan University of Medical Sciences, Hamadan, Iran

**Keywords:** angiogenesis, biomarker, hypoxia, immunotherapy, tumor vasculature, nanoparticles

## Abstract

Angiogenesis is a hallmark of cancer biology, and neoadjuvant therapies targeting either tumor vasculature or VEGF signaling have been developed to treat solid malignant tumors. However, these therapies induce complete vascular depletion leading to hypoxic niche, drug resistance, and tumor recurrence rate or leading to impaired delivery of chemo drugs and immune cell infiltration at the tumor site. Achieving a balance between oxygenation and tumor growth inhibition requires determining vascular normalization after treatment with a low dose of antiangiogenic agents. However, monotherapy within the approved antiangiogenic agents’ benefits only some tumors and their efficacy improvement could be achieved using immunotherapy and emerging nanocarriers as a clinical tool to optimize subsequent therapeutic regimens and reduce the need for a high dosage of chemo agents. More importantly, combined immunotherapies and nano-based delivery systems can prolong the normalization window while providing the advantages to address the current treatment challenges within antiangiogenic agents. This review summarizes the approved therapies targeting tumor angiogenesis, highlights the challenges and limitations of current therapies, and discusses how vascular normalization, immunotherapies, and nanomedicine could introduce the theranostic potentials to improve tumor management in future clinical settings.

## 1 Introduction

Rapid growth and progression of solid tumors results in hypoxia, which drives tumor angiogenesis to remove waste products and provide nutrients and oxygen to support tumor growth and survival (1). Under hypoxic conditions, activated hypoxia-inducible factor-1 (HIF-1) upregulates vascular endothelial growth factors (VEGFs), which bind to their receptors (VEGFRs), expressed on endothelial cells (ECs) or tip cells in established vessels, followed by remodeling of the surrounding extracellular matrix (ECM) and formation of new blood vessels (1). Tumor-associated blood vessels are characterized by abnormal structure and morphology, immature basement membrane, excessive branching, discontinuous EC junctions resulting in high permeability, and resistance to senescence (2). Cancer cells, regulatory immune cells [e.g., immature dendritic cells (DCs), regulatory T cells (Tregs), myeloid-derived suppressor cells (MDSCs), and tumor-associated macrophages (TAM)], and stromal cells also secrete the various cytokines and proangiogenic factors that support tumor angiogenesis (3). In summary, circulating tumor cells promote metastasis, and angiogenesis facilitates cell proliferation, tumor progression, and migration of invasive types into the bloodstream. Therefore, modulation of tumor vasculature may be a promising alternative therapeutic approach in combination with other standard therapies such as immunotherapy and radio/chemotherapy.

We have focused on immunotherapy, and monoclonal antibodies (mAbs) targeting VEGF/VEGFRs have been developed, leading to FDA approval and improvement in overall survival (OS) in some patients treated with mAbs (4). However, drug resistance and hypoxia induction minimize the clinical benefit of anti-angiogenic therapy in some cancers (5) and, more importantly, disruption of the vasculature itself compromises the therapeutic efficacy of chemotherapy or radiotherapy, which are often used in combination with immunotherapy. Although the tumor microenvironment (TME) and multiple cellular and molecular mechanisms provide immunosuppressive processes and impair the host’s anti-tumor immune surveillance (6), complete disruption of the tumor vasculature also interferes with the clinical benefit of immunotherapeutic approaches such as adoptive cell therapy (ACT), which require infiltration of blood vessels into the tumor (7). In addition, many drugs and immune cells require normal oxygen levels to activate and kill cancer cells (8). Considering these challenges, a promising alternative therapy is the remodeling of the tumor vasculature or vascular normalization (9), which overcomes the hypoxic TME (10) and provides the condition for the administration of immunotherapy (8), chemotherapy (11), and radiotherapy (12). For example, vascular normalization improve the efficacy of administered immune checkpoint blockade (ICB) therapy (anti-PD1/PDL1 mAbs), which causes serious immune-related adverse events (8).

Herein, we briefly review existing monotherapies targeting tumor angiogenesis, discuss the limitations and challenges within these therapies, and then provide a perspective on approaches including TME reprogramming approaches, combination therapy, and nanoimmunotherapy that may improve the therapeutic efficacy of vascular-based immunotherapies.

## 2 Targeted therapy for tumor angiogenesis

Since the discovery of angiogenesis as a major driver of tumor growth and progression, various anti-angiogenic agents have been developed in the form of monotherapy immunotherapies and chemotherapies or their combinations, and some of them such as bevacizumab, ramucirumab, trastuzumab and pertuzumab have been approved by the FDA for human cancer therapy (**Table 1**). These therapies include biotherapeutic mAbs that target the ligand-receptor interaction and small molecule tyrosine kinase inhibitors (TKIs) that target the kinase activity of receptor TKs to inhibit downstream signaling cascade activation (34). TKIs and mAb are commonly developed against VEGF/VEGFR, epidermal growth factor receptor (EGFR), and fibroblast growth factor-2 (FGF-2) and their related signaling pathways. FGF-2 has proangiogenic behaviors in paracrine effect and by induction of EC proliferation and migration and promotes angiogenesis by inducing secretion of MMPs to remodel ECM (35). Platelet-derived growth factor (PDGF)-BB (36) and angiopoietins (ANGPTs) (37) are other growth factor molecules that promote vascular maturation and stabilization of newly formed vessels and have been developed for targeted tumor angiogenesis. However, antiangiogenic monotherapy has provided more benefit in highly angiogenesis-dependent tumor types such as advanced renal cell carcinoma (RCC), hepatocellular carcinoma (HCC), and colorectal cancer (CRC) (13) (15, 16). Therefore, it is necessary to combine these therapies (i.e., mAbs and small-molecule drugs targeting VEGF, EGFR, and PDGFR; **Table 1**) or to combine them with radiotherapy to improve survival in patients with other tumors or combined with radiotherapy to improve OS in patients with other tumors (29, 30) (31). In fact, monotherapy with antiangiogenic agents sometimes resulted in vascular depletion but not normalization, followed by induction of hypoxia, nutrient-deprived effector immune cells, drug resistance, and increased tumor invasiveness (5) (8). Thus, the introduction of the “window of vessel normalization” would lead to better outcomes in the clinic. However, depending on the tumor type and the dose of antiangiogenic agents, this window time would be different and could be optimized and extended by various strategies to improve therapies targeting tumor angiogenesis (Section 4).

**Table 1:** Examples of FDA-approved therapies that target tumor angiogenesis.

Therapy/agent (s)	Target	Disease	Refs
Immunotherapy (mAb, monoclonal antibodies)			
Bevacizumab (Avastin®) (humanized pan-anti-VEGF-A mAb)	VEGF-A	Colorectal cancer, non-small-cell lung cancer, cervical cancer, ovarian cancer, renal cell carcinoma, glioblastoma	(13)
Ramucirumab (fully human IgG1 mAb)	VEGFR-2	gastric or gastro-oesophageal junction cancers, colorectal cancer, hepatocellular carcinoma, non-small-cell lung carcinoma	(14)
Cetuximab (recombinant chimeric human/mouse IgG1 mAb) Panitumumab (IgG2 kappa mAb)	EGFR	Colorectal cancer	(15, 16)
Trastuzumab and Pertuzumab (humanized IgG1 mAb)	HER2	HER2-positive breast cancer	(17)
Immunomodulatory agents (Thalidomide and Lenalidomide)	TNF-α, ILs, IFNs, VEGF, bFGF	Multiple myeloma	(18, 19)
Chemotherapy (small-molecule drugs)			
Gemcitabine	EGFR	Squamous non-small-cell lung cancer	(20)
Sorafenib Sunitinib (Sutent®) Lenvatinib (Lenvima®) Imatinib (Gleevec®) Pazopanib Regorafenib	VEGFR-1, VEGFR-2, VEGFR-3, PDGFR family, RAF	Hepatocellular carcinoma, renal cell carcinoma, thyroid cancer	(21–24)
Gefitinib	EGFR	Non-small-cell lung cancer	(25)
Neratinib Lapatinib Afatinib	EGFR-HER-2	HER-2 positive breast cancer and non-small-cell lung cancer	(26–28)
Combinatorial therapy			
Erlotinib plus gemcitabine	EGFR	Pancreatic adenocarcinoma	(29)
Cisplatin plus Necitumumab	EGFR	Squamous non-small-cell lung cancer	(20)
Bevacizumab plus oxaliplatin	VEGF-A	Colon cancer	(30)
Cetuximab plus Radiotherapy	EGFR	Squamous cell carcinoma of the head and neck	(31)
Bevacizumab plus irinotecan, fluorouracil, and leucovorin	VEGF-A	Metastatic colorectal cancer	(32)
FOLFIRI plus cetuximab *versus* FOLFIRI plus bevacizumab	EGFR and VEGF-A	Metastatic colorectal cancer	(33)

## 3 Challenges in modulating tumor angiogenesis

**Figure 1:**
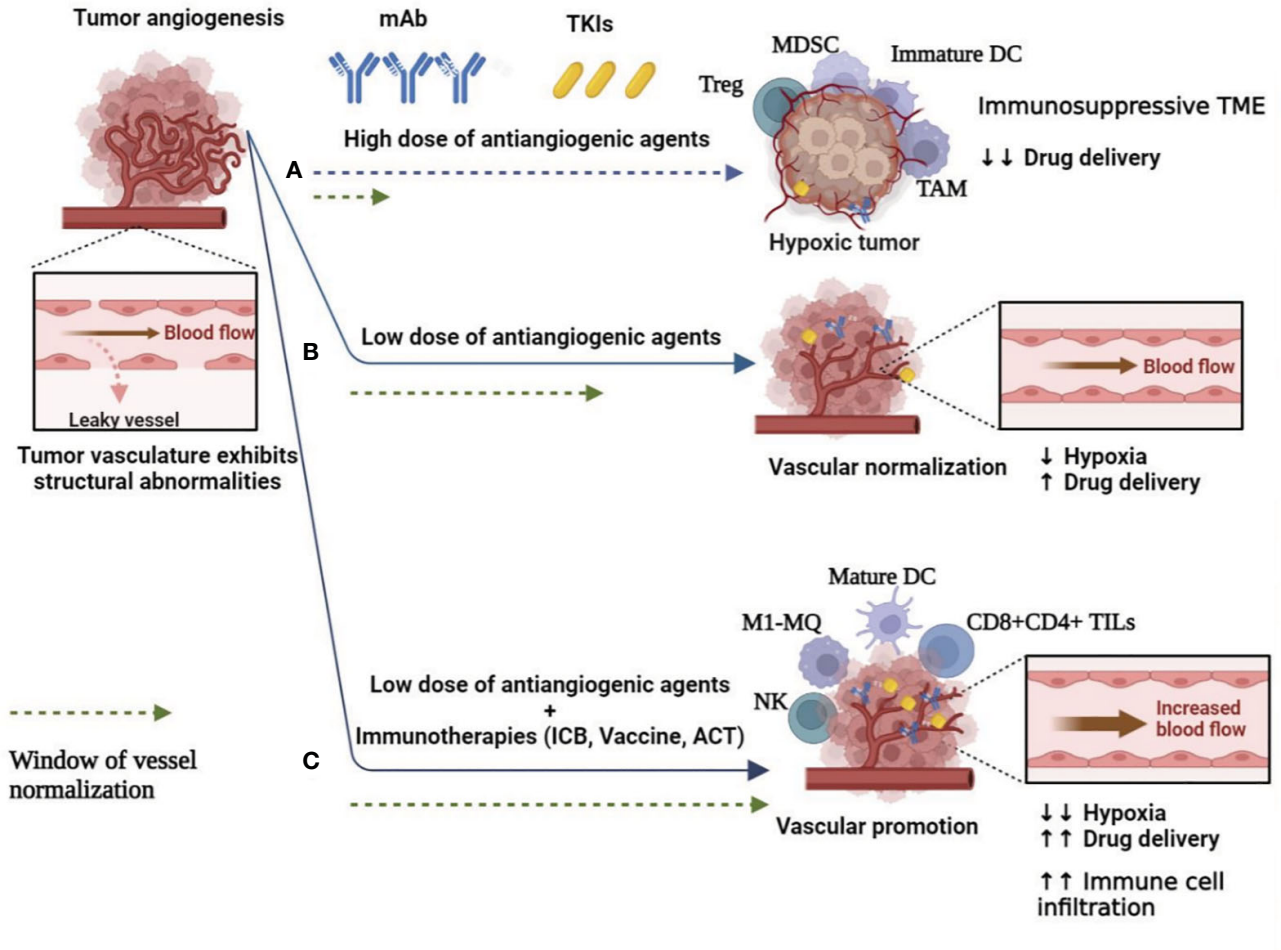
Switching the abnormal and leaky tumor blood vessels to normal function to improve angiogenesis-based cancer therapies. **(A)** High dose of antiangiogenic agents (mAb and TKIs) resulted in complete vascular disruption and hypoxia, and immunosuppressive TME, which support cancer cell survival, limit drug delivery and induce drug resistance. Furthermore, the abnormal TME is associated with increased infiltration of immunosuppressive Tregs, TAMs, MDCSs, and immature DCs. Hypoxic conditions also impair the therapeutic efficacy of radiotherapy and chemotherapy (6, 38, 39). **(B)** Low doses of antiangiogenic agents lead to transient restoration of blood flow and perfusion. This reduces hypoxic TME and increases drug delivery to tumor sites (40). **(C)** Anti-angiogenic agents combined with immunotherapies could expand the normalization window, enhance drug delivery and immune effector cell infiltration, and thus reprogram the immunosuppressive tumor microenvironment to an immunosupportive one (41). DC, dendritic cell; MQ, macrophage; mAb, monoclonal antibodies; MDSC, myeloid-derived suppressor cells; TAMs, tumor-associated macrophages; TKI, tyrosine kinase inhibitor; TIL, tumor infiltration lymphocyte; TME, tumor microenvironment; Tregs, regulatory T cells.

In addition to hypoxia and niche modulation of effector immune cells, targeting angiogenesis resulted in drug resistance derived from TME components such as stromal cells, progenitor cells, and regulatory immune cells (Tregs, MDSCs, and TAMs) that support tumor progression and immune escape (6, 38, 39) (**Figure 1A**). Blockade of vascularization leads to ECM remodeling, the release of proinflammatory/proangiogenic factors (42), induction of signaling pathways that mediate EC proliferation (43), and stimulation of proangiogenic myeloid cells (44), followed by recruitment of endothelial progenitor cells (EPCs), angiogenesis, and resistance to anti-VEGF therapy. Cancer-associated fibroblasts (CAFs) are the most common type of stromal cells in the TME that reprogram ECs and produce MMPs to degrade ECM and promote angiogenesis (45). Importantly, high-dose anti-VEGF therapy plus hypoxic nicotine results in a population of cancer stem cells (CSCs) that are responsible for drug resistance, tumor relapse, and metastasis (6, 46). In addition, the heterogeneity of blood vessels in different organs or tissues has complicated anti-VEGF therapies in solid tumors (47).

Disruption of the blood supply acts as a double-edged sword, impairing effector T-cell infiltration, limiting drug delivery to tumor sites (**Figure 1A**), and depriving radiotherapy of the oxygen needed to generate cytotoxic radical ions, all of which reduce antitumor efficacy (5, 8). Cancer cells can potentially differentiate into endothelial-like phenotypes that support neoangiogenesis in an EC-independent manner and are responsible for patients’ resistance to blockade of the VEGF pathway (48). In addition, VEGF blockers induce the production of proangiogenic factors (e.g., VEGF, bFGF, and PDGF), which drive compensatory mechanisms of promoting angiogenesis and disease progression (49), emphasizing the development of a new generation of drugs to simultaneously target the VEGF signaling pathway and alternative angiogenic derivers.

In terms of function and structure of the approved biotherapeutic agent, after intravitreal drug injection, the full size of mAb limited their kidney excreted through urine and the Fc domain of mAb bind to neonatal Fc receptor (FcRn), expressed by ECs, resulting in prolonged systemic exposure as well as possible adverse events (50). Unlike mAbs that bind specific targets, multi-targeted TKIs have broader bioactivity and exhibit greater antitumor activity but are more toxic (51). In addition, productive anti-VEGF agents need to be administered frequently, which asides from drug resistance and commercially present a high cost of treatment that has limited their widespread use. Therefore, validated and sensitive biomarkers and gene expression signatures can monitor response to therapies and predict side effects and tolerability of routine clinical use.

According to the type of anatomical tumor types and *in vivo* biological parameters, mAb have different pharmacokinetics, which affected their performance and selection for clinical application (52). Considering the safety profile of mAb, advanced biotechnological tools have developed single-chain antibody fragment (scFv)–based anti-VEGF antibodies or Fab-based agents such as Aflibercept and Conbercept that combine the Fc portion of a complete mAb to two highest affinity domains of VEGFR1 and VEGFR within high affinity for VEGF isoforms (52). Moreover, nanotechnological tools encapsulating of BioDrugs are being developed to extend their half-life *in vivo* or combine them with other therapeutic agents with different mechanisms of action to reduce the frequency and dose of intravitreal injections and minimize undesired systemic exposure, preserved mAb bioactivity, and controlled drug release in tumor sites (53).

## 4 Normalizing tumor vasculature improves cancer immunotherapy

The limitation of complete vascular occlusion suggests that restoring some degree of vascular perfusion capacity could increase the penetration of therapeutic cells into the tumor and improve the delivery of chemotherapeutics or provide sufficient oxygen to enhance the efficacy of radioactive agents. Considering the short and revisable normalization window and the dose-dependent effect, the low dose of antiangiogenic agents can provide efficient and durable vascular normalization (**Figure 1B**). When anti-VEGF treatment is used at low doses, blood vessel function is improved by reorganizing the pericyte lining to reduce vascular permeability and hypoxia (40). Prolonging the normalization window also improves the antitumor immune responses by increasing effector T-cell infiltration (54), ECM remodeling (55), and reprogramming of immunosuppressive TME to enhance the therapeutic efficacy of cancer immunotherapies (39, 54), including immune checkpoint blockers (ICBs) by blocking immune checkpoints, programmed death-ligand 1 (PD-L1), and PD-L2 in tumor cells and cytotoxic T-lymphocyte–associated protein 4 (CTLA-4) and programmed cell death protein 1 (PD-1) expressed on T cells (56). Interestingly, depletion of ICBs and Tregs promotes vascular normalization by reducing tumor vascular density and reactivating IFNγ producers (i.e., CD8+/CD4+ effector T cells), which IFNγ has the potential to limit tumor angiogenesis (54). In addition to immune suppression, Tregs expressing VEGFRs, such as neurophilin-1 mediated angiogenesis and anti-VEGF mAb, can lead to Tregs depletion and enhance the immunologically beneficial effects in the TME. VEGF also suppresses DC maturation and upregulates the expression of PD-L1 on DC and PD-1 and CTLA-4 on T cells, leading to T-cell exhaustion and suppression of their function, resulting in immunosuppression (57) (**Figure 1A**). Therefore, targeting VEGF/VEGFR-enhanced anti-tumor immunity and tumor cell elimination.

Another strategy to induce vascular normalization is *in situ* delivery of angiostatic factors such as tumor necrosis factor alpha (TNFα), which at low doses can stabilize the tumor vasculature and improve vascular permeability (58). Moreover, proangiogenic signaling induced by angiopoietin 2 (ANG2)/TIE2 cytokines contributes to vascular VEGF-dependent angiogenesis and increases microvessel density of the TME as well as vascular permeability, and thus, dual targeting of ANG2 and TIE2 or ANG2 and VEGF extends both the window of normalization and reduces metastatic dissemination in patients with glioblastoma compared with VEGF or ANG2 inhibition alone (39, 59). Depending on the cell source, IFNγ produced by T cells limits the tumor angiogenesis (54), while IFNγ-expressing ECs upregulate PD-L1 in tumor cells and limit antitumor immunity, which guided that multi-targeting of ANG2, VEGF, and PD-L1 enhanced antitumor responses in transplanted tumor models (57, 60). Like IFNγ, the anti–VEGF-ANG2 antibody upregulates immune inhibitory molecules such as PD-L1 on both ECs and tumor cells to promote T-cell exhaustion, highlighting the benefit of co-treatment with anti–PD-1 antibody (61). Tumor type also determines the benefits of dual VEGF-ANG2 inhibition that in lymphoid and myeloid cell populations infiltration of CD8+/CD4+ effector T cells and reprogramming of M2-like TAM to M1-like one with an anticancer phenotype, respectively, contribute to the therapeutic efficacy of dual antiangiogenic therapy (8, 39, 59).

It requires accumulation of CD8+ effector T cells in tumor sites before exerting its antitumor activity. In non-inflamed TME and highly angiogenic tumor state, bFGF/VEGF activities influence ECs toward decreased expression of endothelial adhesion molecules (EAMs), including E-selectin, ICAM1, and VCAM1, thus hindering leukocyte homing, extravasation, and infiltration into tumor tissue (62). Thus, the combination of antiangiogenic agents and immunotherapies switch cold TME to hot one, which improves the infiltration and functions of T cells in tumors (such as metastatic RCC and breast cancer) (**Figure 1C**), especially with a high incidence of high endothelial venules-expressing ICAM1 and VCAM1 to overcome the physical barrier for circulating T cells (41). Preclinical studies have shown that administration of low doses of an anti-VEGFR2 antibody increases CTL infiltration and thus improves the anticancer efficacy of subsequent therapies by tumor vaccination or ACT approaches (56, 63). In terms of cell therapy, nanobody (VHH)-based CAR T cells were engineered to target VEGFR2-expressing tumor cells and were considered as a candidate for ACT-based immunotherapy of solid tumors (64). Like PD-L1, tumor cells and tumor-associated ECs overexpress a protein called galectin 1, which induces apoptosis and exhaustion in resting T cells, limiting their infiltration and function (65). Therefore, the anti-tumor immune activity targeting galectin 1 synergized with anti-PD-L1 antibodies. Combinatorial therapy also reprograms the TME through oxygenation and reduction of hypoxia, tissue perfusion, and interstitial fluid pressure, complementing existing standard therapies. Overall, combinations of antiangiogenic agents and immunotherapy (ICBs, vaccine, and ACT) provide an update on the current clinical immunotherapies and currently being tested with encouraging results or ongoing, and some of them, especially combinations of antiangiogenic agents and ICBs approved in the clinic (**Table 2**).

**Table 2:** Various combinations of anti-angiogenic and immunotherapeutic agents.

Antiangiogenic agent	Combined with:	Cancer type	Status	Trial no:	Refs:
ICBs					
Bevacizumab	Atezolizumab (anti-PD-L1 mAb) + carboplatin+ paclitaxel	Advanced-stage non-squamous NSCLC	Phase III FDA approved	NCT02366143	(66)
Axitinib (kinase inhibitors)	Pembrolizumab (anti-PD1 mAb)	Advanced-stage RCC	Phase III FDA approved	NCT02853331	(67)
Axitinib	Avelumab (anti-PD1 mAb)	Advanced-stage RCC	Phase III FDA approved	NCT02684006	(68)
Lenvatinib (multiple TKI)	Pembrolizumab	Advanced-stage solid tumors	Phase III FDA approved	NCT02501096	(69)
Bevacizumab	Atezolizumab	metastatic HCC	Phase III FDA approved	NCT03434379	(70)
Bevacizumab	Atezolizumab	Metastatic CRC	Phase III Recruiting	NCT02997228	(71)
Vanucizumab	Atezolizumab	Ovarian, primary peritoneal cancer	Phase I Completed	NCT01688206	(72)
Bevacizumab	Ipilimumab (anti- CTLA-4 mAb)	Melanoma	Phase I Active, not recruiting	NCT00790010	(73)
Cell therapy					
Sunitinib poly-ICLC ± KLH	Intuvax (allogenic cell-based therapy)	mRCC	Phase II: active, not recruiting	NCT02432846	(74)
Sunitinib	autologous DC immunotherapy	Advanced RCC	Phase II	NCT00678119	(75)
DC101 (anti-VEGFR2 mAb) and B20 (anti-VEGF mAb)	Anti-PMEL T cells, PMEL vaccine, and IL-2	Melanoma	Preclinical	–	(76)
Anti-VEGF mAb	Tumor-associated peptide-pulsed DCs	Sarcoma	Preclinical	–	(77)
Recombinant human endostatin	Cytokine-induced killer cells (CIK cells)	lung carcinoma	Preclinical	–	(78)
Bevacizumab	GD2-CAR T cells	Neuroblastoma	Preclinical	–	(79)
Bevacizumab	allogeneic NK immunotherapy	Solid tumors	Phase I/II	NCT02857920	
Cancer vaccines					
Bevacizumab	Peptide vaccine (EGFRvIII, EphA2, Her2/neu peptide)	Glioblastoma	Phase II: active, not recruiting	NCT02754362	(80)
Bevacizumab	HSPPC-96 (personalized peptide-based vaccine)	GBM	Phase II: active, not recruiting	NCT01814813	(81)
Sunitinib Tremelimumab	PF-06755990 (adenovirus expressing PSA, PSMA, and PSCA)	Prostate cancer	Phase I: recruiting	NCT02616185	(82)
Sunitinib	IMA901 (multipeptide cancer vaccine)	mRCC	Phase III completed	NCT01265901	(83)
Sunitinib	DC-based vaccine expressing IL-12 and pulsed with OVA-peptide (DC-IL12-OVA)	B16-OVA tumor model	Preclinical	–	(84)
Aflibercept (Eylea®) (VEGF trap)	Tumor-antigen-specific picornaviral vaccination	Glioblastoma	Preclinical	–	(85)
DC101 (anti-mouse VEGFR2 mAb)	Whole tumor cell vaccine (mitomycin treated and GM-CSF secreting)	Breast cancer	Preclinical	–	(56)
Adenoviral- expression of sVEGFR1 and sVEGFR2	Whole tumor cell vaccine (GM-CSF secreting)	Colon cancer Melanoma	Preclinical	–	(86)
Sorafenib (VEGFR TKI)	Pox virus vaccine expressing CEA and three co-stimulatory molecules	Colon cancer	Preclinical	–	(87)

Although combinations of anti-angiogenic and cancer vaccines have shown promising results in preclinical models, these combinations have not yet been recapitulated in humans (88). Prior to clinical application, identification of dose-limiting toxicities and maximum tolerated doses of the drug combination are important considerations and highlight the importance of identifying potential biomarkers that can predict effective doses of treatment responses.

## 5 Nanomaterials improve antiangiogenic cancer immunotherapy

**Figure 2:**
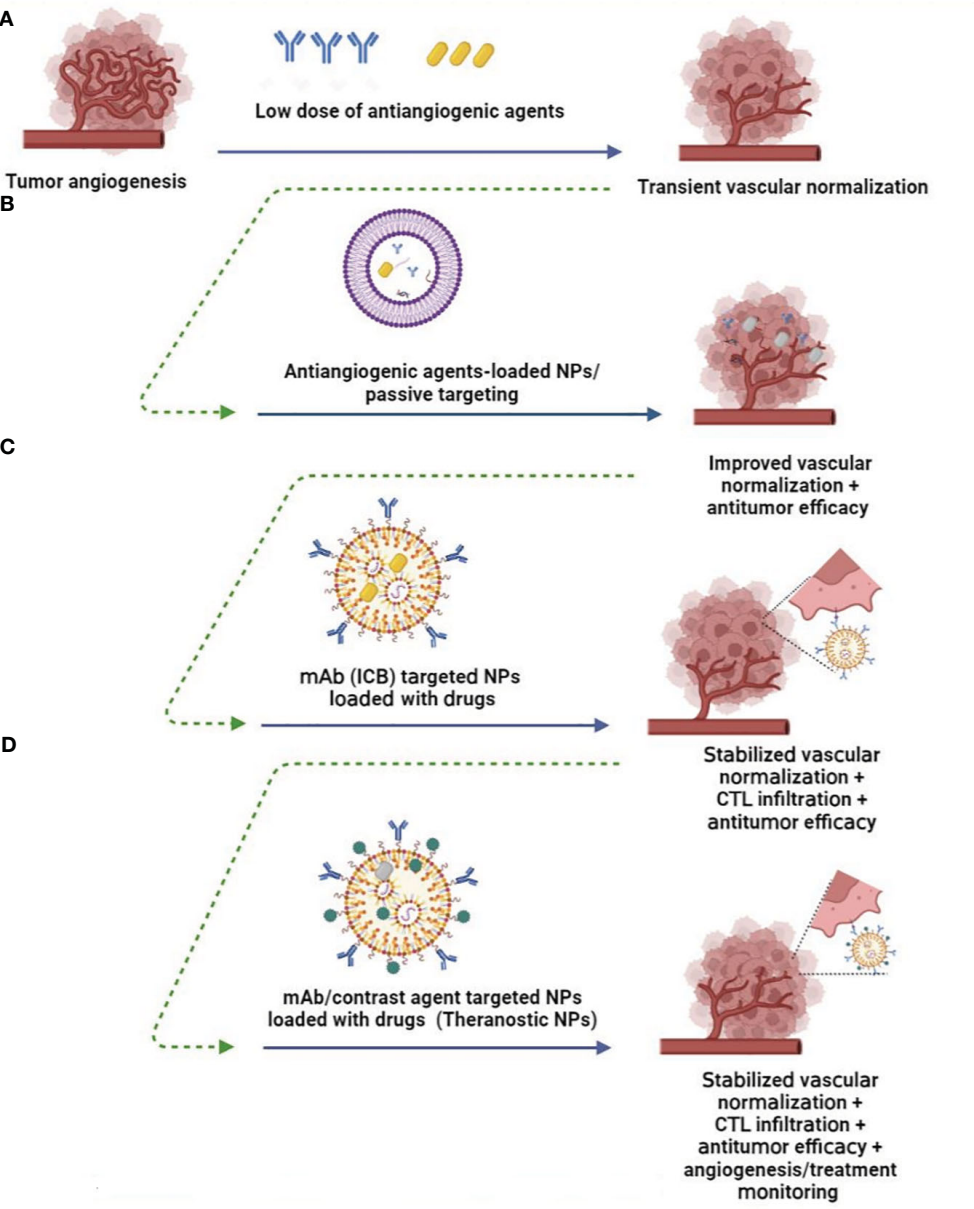
Nanomedicine improves angiogenesis-based cancer therapy. **(A)** Low doses of antiangiogenic agents induce transient normalization and reduce hypoxic condition (9). **(B)** Nanoparticles (NPs) loaded with lower doses of anti-angiogenic agents enhance their delivery while protecting them *in vivo*, provide passive targeting, and promote the EPR effect while promoting vascular normalization (89). **(C)** Therapeutic ligand-targeted NPs (e.g., mAb) deliver drugs to specific tumor cells, prolong vascular normalization, and induce antitumor immune responses through CTL infiltration or reprogramming of the immunosuppressive TME (90). **(D)** mAb-targeted theranostic NPs provide antitumor efficacy, while conjugated imaging probes enable angiogenesis visualization, monitoring of therapies, and determining the optimal dosage of therapeutic agents *in vivo* for clinical setting of vascular normalization and tumor eradication (91).

ICBs are known to cause immune-related adverse events, some of which are severe. Although vascular normalization could improve the efficacy of immune checkpoint inhibitors and overcome resistance to cancer immunotherapy (61), this companion yet occurs transiently (**Figure 2A**) and resulted in acute toxicity grading including hematological and liver function abnormalities (92). Therefore, neoantigen load of tumors in cancer vaccine or delivery of ICBs or antiangiogenic agents within nano platforms or production of nano-sized agents hope to overcome a barrier to effective immunotherapy and elicit the most potent antitumor responses. In addition, passive and active targeted nanomaterials have reduced the frequency of intravitreal injections. It minimizes unwanted systemic exposure by enhancing the binding of NPs to tumor cells, reducing their non-specific uptake, provided passive accumulation of drugs in tumor tissue is called the EPR effect (93) (**Figure 2**).

Anti-angiogenic nanoagents inhibit neoangiogenesis directly and indirectly by targeting ECs and signaling pathways that support tumor angiogenesis (**Figure 2B**). Recombinant human endostatin (Endostar/rhES) mediates vascular disruption by targeting VEGF-induced angiogenesis and has been used in the clinical setting to treat non-small-cell lung cancer. To improve *in vivo* efficacy and reduce dosing frequency, PEGylated gold (Au)NPs loaded with rhES were developed. Results indicate that rhES-AuNPs-PEG reduced vascular permeability and improved vascular normalization. Subsequently, this nanosystem improved the intratumoral delivery and antitumor efficacy of cytotoxic 5-FU in H22 tumor-bearing mice (94). Folic acid-modified Au NPs induced tumor vessel normalization by upregulating EC-VE-cadherin, which mediates tight junctions between pericytes. This was followed by improved vascular perfusion and accumulation of CD3+ CD8+ T cells in the TME (95). Taking into account the specific pH, temperature and enzymes formed by TME, some TME-responsive nanomaterials have been developed. For example, pH-sensitive delivery system selenium (Se) NPs loaded with anti-VEGF siRNA significantly accumulated in the tumor region and reduced tumor growth *in vivo* mediated by *VEGF* gene silencing (96).

The surface of NPs could be modified by targeting ligands (e.g., peptides, antibodies, and aptamers) to increase the accumulation of NPs at the tumor site and provide more efficient targeted therapy (**Figure 2C**). For example, APT_EDB(fibronectin extra domain B)_ peptide-conjugated PEG-PLA (polylactide) NPs loaded with paclitaxel can target fibronectin on both neovascular and glioma cells to exert its antiangiogenic therapeutic effect and inhibition of tube formation by ECs, while inducing antitumor activity by delivered paclitaxel (97).

However, due to the complexity of tumor angiogenesis, targeting only a single factor may lead to drug resistance, which could be addressed by targeting NPs to deliver multiple agents. PEGylated Ag_2_S quantum dots (QDs)-NPs surface modified by cRGD peptide and loaded with an antiangiogenic agent (TNP-470) and doxorubicin (DOX), T&D@RGD-Ag_2_S, showed long-lasting blood circulation and highly specific vascular binding, resulting in accumulation at the tumor site and inhibited angiogenesis and tumor growth in a human glioma xenograft model (98). NPs can also realize the combined treatment of antiangiogenic agents with photothermal therapy (PDT). For example, dual-functional liposomes containing near-infrared (NIR) dye (IR780) and sunitinib inhibited angiogenesis-mediated tumor growth *in vivo* after sunitinib was released from NPs by laser irradiation, and further, this platform induced tumor cell killing upon PDT mediated by IR780-loaded liposomes (99). Biocompatibility that reduces toxicity is a key biological requirement for developed NPs, which could be achieved by biosynthesizing NPs or using natural biomolecules or cells as biomimetic drug delivery systems. For example, RBCs were decorated with RGD peptide and then co-loaded with DOX and NIR dye indocyanine green bound to bovine serum albumin, IB&D@RBC-RGD, which demonstrated the synergistic therapeutic effect of combined PPT and chemotherapy, thus showing significant antitumor activity (100).

With the combination of anti-angiogenic agents and immunotherapy, NPs can also restore tumor vasculature to promote the infiltration of anti-tumor immune cells into the immunosuppressive TME and reduce immune-related side effects caused by ICB (**Figure 2B**). Micellar NPs encapsulated with sunitinib in the combination of lipid-coated calcium phosphate (LCP) NPs containing tyrosinase-related protein 2 (Trp2) peptide and CpG ODN, LCP-Trp2 vaccine, increased CTL infiltration and decreased the number and percentage of MDSCs and Tregs in the TME of an advanced murine melanoma model (90). Nitric oxide (NO)–based NPs provide sustained NO release in TME and HCC that reprogrammed the immunosuppressive TME, as evidenced by downregulation of PD-L1 expression and inhibition of M1 macrophage polarization to M2 phenotype, all of which improved the therapeutic effects of cancer vaccines (101). NPs decorated with anti-PD-L1 and 4-1BB antibodies simultaneously blocked PD-L1 pathway-mediated CTL apoptosis and reactivated them by inducing the 4-1BB co-stimulatory pathway in CTLs (102). Since tumor ECs express 4-1BB, these immunoswitch NPs can stimulate ECs to express adhesion molecules (i.e., ICAM-1, VCAM-1, and E-selectin) required for CTL infiltration (103). Combination therapy of engineered platelets loaded with anti-PDL1 mAb (P@aPDL1) and vadimezan, as an inducer of local tumor bleeding, led to the development of an off-the-shelf cell therapy and biomimetic carrier that recruits platelets to the hemorrhagic tumor site and locally enhances the accumulation and release of aPDL1 to elicit T-cell–based immunotherapy in a metastatic breast tumor model (104). Recently, a combination of radiotherapy, liposomal irinotecan with radiosensitizing properties, apatinib (TKI targeting VEGFR-2) and PD-1 antibody was evaluated in a phase II clinical trial that enrolled patients with advanced solid tumors and showed promising tolerability and anti-tumor activity (Clinical Trial ID: NCT04569916). Taken together, the application of multifunctional nanocarriers for delivery of anti-angiogenic agents in combination with approved immunotherapies may induce lower interstitial fluid pressure, increased intratumoral oxygenation, reduced drug dosage and resistance, and increased accumulation of BioDrugs or promoted trafficking of immune cells within the tumor site.

Finally, it is noteworthy that nanomaterials carrying diagnostic or imaging agents also allow non-invasive visualization of tumor angiogenesis, monitoring of response to anti-angiogenic therapies and, more importantly, determining the optimal dosage of therapeutic agents (105) (**Figure 2D**). Targeting VEGFR and ECs has been proposed for targeted imaging of tumor angiogenesis using CT, PET, SPECT, MRI, optical imaging, and ultrasound imaging. However, these imaging techniques present too low resolution for microvascular imaging, which could be improved by nano-sized agents or NPs containing low-dose antiangiogenic drug-mediated tumor vessel, tissue on, and tissue perfusion and oxygenation. In addition, an RGD-functionalized nanocarrier has been used as a contrast agent that allows the detection of ongoing angiogenesis. For example, cyclized asparagine-glycine-arginine peptide (cNGR) peptide surface functionalized of Au NPs targets aminopeptidase-N on the endothelium and provides imaging using computed tomography (CT) in 4T1 xenograft model (91). Considering the role of MMPs in ECM degradation and angiogenesis promotion, Ryu et al. developed MMP-responsive nanoprobes decorated with nanoparticulted human serum albumin that exhibited prolonged circulation half-life and enhanced fluorescence for optical imaging emission during angiogenesis in a mouse hindlimb ischemia model (106). Biomedical NPs such as carbon-derived quantum dots are other promising candidates that have been used for molecular imaging of tumor angiogenesis *in vivo* (93).

Theranostic NPs (e.g., mesoporous silica NPs and Au NPs) could act as anti-angiogenesis agents and tumor vascular normalizers even in a drug-free manner, which subsequently this tumor vascular normalization improves the delivery efficiency of nanotheranostic agents. Moreover, the fluorescence recovery after the release of DOX (a chemo drug with fluorescent property) from the liposomal formulation can be used as an indicator to monitor the chemotherapeutic progress (107). Nanosized iron oxide NPs with their intrinsic imaging capabilities are exquisitely suitable and even approved (e.g., ferumoxsil) for theranostic applications. pH-degradable bovine serum albumin-functionalized Fe_3_O_4_ NPs and loaded with lenvatinib was able to induce vessel normalization and modulate immunosuppressive TME of HCC while Fe_3_O_4_ provided *in vivo* visualization tracking by magnetic resonance imaging (MRI) and also magnetic particle imaging (108). Combined, nanotechnology has and will continue to maximize the clinical benefits of existing therapies targeting tumor angiogenesis. Although NPs are potent, their intrinsic efficacy must be optimized by determining their various parameters, including safety, size, shape, wettability, charge, and effective concentration (53). In summary, therapeutic agent NPs with the function of vascular normalization inducers to achieve specific targeting of solid tumors while exerting efficient antitumor effects *in vivo*.

## 6 Conclusion

Here, we summarize the recent developments in integrated therapies of antiangiogenic agents, immunotherapy, and nanotechnology for tumor angiogenesis. An in-depth understanding of the angiogenesis processes, selective biomarkers, and immunoregulatory factors involved in the TME of solid cancer tumorigenesis, especially in metastatic tumors, may allow the development and application of efficient cancer therapies in the future. To date, the vast majority of vascular remodeling drugs or contrast agents used in routine monitoring are administered systemically, causing off-target effects in healthy tissues. The development of surface-modified nanosystems has provided more robust and durable normalization strategies to widen the therapeutic window while allowing monitoring of therapies and changes in the TME vasculature. In addition, nanotherapeutics address the challenges of timing and dosing of vascular remodeling agents or combinatorial therapies. They could become part of the treatment approach for advanced solid tumors.

## Author contributions

HD-M: Conceptualization, Investigation, writing – original/revised draft, Writing - review & editing, Visualization, Supervision, and Project administration. RN, MKe, and MA: Investigation, writing – original/revised draft. RS and MKa: Investigation, Writing - review & editing. All authors have read and agreed to the published version of the manuscript.
